# Global research dynamics in the Mediterranean diet and diabetes mellitus: a bibliometric study from 2014 to 2024

**DOI:** 10.3389/fnut.2024.1480856

**Published:** 2024-11-14

**Authors:** Yuanyuan Yan, Zonghuai Li, Yuanchu Lian, Pingping Liu, Bo Zhang, Juan Chen

**Affiliations:** ^1^Department of Pharmacy, Sanya Central Hospital (The Third People's Hospital of Hainan Province), Hainan, China; ^2^Scientific Research Center, Guilin Medical University, Guilin, China

**Keywords:** bibliometric analysis, insulin sensitivity, antioxidant, gut microbiota, chronic disease prevention

## Abstract

**Objective:**

The Mediterranean diet (MedDiet) has been found to have benefits for diabetes mellitus (DM), but a bibliometric analysis of its association with DM has yet to be conducted. This paper aims to explore the current status and research hotspots on the connection between the Mediterranean diet and DM from 2014 to 2024, providing a reference for future studies.

**Methods:**

We retrieved articles published between 2014 and 2024 from the Web of Science database and analyzed them using R software, VOSviewer, and CiteSpace.

**Results:**

A total of 2,806 articles were included in this study. Research on the relationship between the MedDiet and DM showed a steady increase in publication volume from 2014 to 2019, followed by a sharp rise from 2020 to 2023. Spain was the leading country in terms of publication volume, followed by Italy, the United States, China, and Greece. Spain also led in international collaborations, with CIBER—Centro de Investigación Biomédica en Red and Harvard University being the most prominent collaboration centers. Nutrients was the most frequently published and cited journal in this field. Common keywords in this literature included components such as olive oil, legumes, and red wine. Mechanisms studied in this field primarily focused on antioxidant effects, improvements in insulin sensitivity and secretion, regulation of lipid metabolism, and modulation of gut microbiota.

**Conclusion:**

Research on the beneficial effects of the MedDiet on DM patients has garnered significant attention from researchers worldwide, and it is expected to become a major focus for future DM prevention and treatment. This study provides a comprehensive analysis of the current status and research hotspots regarding the relationship between the MedDiet and DM, offering valuable references for future research.

## 1 Introduction

Diabetes mellitus (DM), as a prevalent and serious chronic disease (1), has a profound impact on individuals and families worldwide (2). It is not only one of the leading causes of adult mortality but is also increasing at an alarming rate. As of 2019, the number of people with DM worldwide had reached 464 million, and this figure is projected to rise to 578 million by 2030. By 2045, it may increase to 700 million (3). The complications of DM can pose fatal threats to patients’ health (4), such as the risk of death from macrovascular complications (5), and the damage to renal cells and microvasculature due to hyperglycemia, which can lead to diabetic nephropathy (6). Additionally, hyperglycemia may trigger the polarization and activation of retinal microglia, potentially causing chronic neuroinflammation and neurodegeneration (7). Conditions such as gastroparesis and diabetic foot are also closely related to DM (8, 9), highlighting the severe threat DM poses to individual health.

Research indicates that lifestyle habits, dietary patterns, and regular exercise are key factors in the development of DM (10). Unhealthy dietary patterns may promote the onset of DM (11). Therefore, researching how to change unhealthy dietary behaviors and promoting healthy dietary patterns that help reduce the incidence of type 2 diabetes is of critical importance (12). In recent years, numerous studies have confirmed the benefits of healthy eating in reducing the incidence of DM (12). Specifically, the Mediterranean diet (MedDiet) has gained attention for its potential benefits in DM prevention (13). The MedDiet, a concept proposed by Ancel Keys (14), is one of the most extensively researched and well-known dietary patterns globally. Originating in the Mediterranean region, this diet is closely tied to the social behaviors and lifestyles of the area. UNESCO has recognized the MedDiet as an intangible cultural heritage, reflecting its deep geographical and cultural roots (15). The MedDiet includes various foods such as extra virgin olive oil, legumes, grains, nuts, fruits, vegetables, dairy products, fish, and wine, which are rich in phytonutrients, especially polyphenols and vitamins, that play a vital role in health (16). Studies have identified key components of the MedDiet, such as olive oil and polyphenols—particularly hydroxytyrosol—as potent agents in cancer prevention through the reduction of oxidative DNA damage (17). Recent bibliometric analyses using tools like CiteSpace have provided insights into the global trends and emerging research hotspots related to the MedDiet. These studies highlight the MedDiet’ s association with the prevention of chronic diseases, including cardiovascular conditions, DM, obesity, and cancer. Additionally, non-alcoholic fatty liver disease has been identified as a growing area of interest (18). The MedDiet’s antioxidant, anti-inflammatory, immunomodulatory, and antimicrobial properties have also been linked to a protective effect against severe COVID-19 outcomes (19). Research on the MedDiet has evolved beyond traditional health benefits, extending into interdisciplinary fields such as neurodegenerative diseases and immunology. In this context, the Mediterranean-DASH Diet Intervention for Neurodegenerative Delay (MIND) diet-a fusion of the MedDiet and the Dietary Approaches to stop Hypertension (DASH) diet-emerges as a promising intervention targeting cognitive health. By prioritizing brain-friendly foods, the MIND diet aims to delay or prevent the onset of neurodegenerative diseases like Alzheimer’s disease (20).

Parallel bibliometric studies have uncovered the multi-dimensional nature of DM research. Key areas include gestational diabetes (21), the role of exercise in DM management (22), and regional research outputs from Middle Eastern countries (23). These studies underscore the intricate relationships between DM and various factors, such as physical activity, pregnancy, and population-specific risks. Although the MedDiet has demonstrated potential in DM prevention through various mechanisms, further research is needed to fully elucidate the underlying pathways.

Although existing research confirms that the MedDiet may prevent DM through various mechanisms, the specific pathways of these mechanisms require further investigation. Therefore, there is still much to explore in the research area concerning the relationship between the MedDiet and DM. Bibliometrics, a discipline that applies mathematics, statistics, and other quantitative research methods to analyze bibliographic information (24, 25), offers advantages over conventional literature review methods. Specifically, bibliometrics can conduct targeted quantitative and qualitative analyses of publications within a specific field, which helps assess the current state, development trends, and emerging frontiers of the research area (26, 27). Additionally, this method allows for comparative analysis of the contributions of different countries/regions, research institutions, academic journals, authors, and researchers.

A significant advantage of high-quality bibliometric research is its ability to highlight the forefront of research, effectively saving time for researchers (28). However, despite the growing number of academic articles on the relationship between MedDiet and DM, there is currently a lack of comprehensive bibliometric studies in this field. As a result, it is challenging to gain a complete understanding of the overall trends, key research focuses, and knowledge evolution trajectories. This study aims to conduct an in-depth bibliometric analysis of research literature published between 2014 and 2024 on the relationship between MedDiet and DM, using analytical tools such as R software, VOSviewer, and CiteSpace. Our primary objective is to explore the evolution of research hotspots in this field over time, identify potential future research directions, and deepen the understanding of existing knowledge as well as the potential applications of the MedDiet in DM. By constructing a macro perspective of current research and identifying trends, differences, and connections in the literature, we aim to provide guidance on potential research directions and emerging areas for future studies.

## 2 Materials and methods

### 2.1 Data collection

The data used in this study were retrieved and downloaded from WoS (Guilin Medical College Edition) on July 18, 2023. The search formula is (TS = (“Mediterranean diet” OR “Mediterranean dietary pattern”) AND TS = (diabetes OR hyperglycemia OR “insulin resistance” OR “glucose intolerance” OR “metabolic syndrome”)) AND (DT = (“Article” OR “Review”)) AND (LA = (“English”)) AND (DOP = 2014-01-01/2024-07-18). Duplicate documents were removed, and the remaining retrieved articles were saved in plain text format and their cited references were exported as complete records.

### 2.2 Data analysis

This study utilized Origin 2018 software to analyze annual publication trends and employed R software (version 3.6.3) with the bibliometrix package (version 4.0) (29), VOSviewer (version 1.6.17) (30), and CiteSpace (version 6.1.4) (31) for bibliometric data analysis and visualization. These tools were selected to ensure the accuracy and reliability of data extraction and analysis processes.

VOSviewer was used to create various visualizations, including co-authorship networks by country and institution, co-citation analysis, and keyword co-occurrence networks. In the co-authorship network construction, only countries with at least five publications and institutions with at least 13 publications were included in the analysis. For co-citation analysis, we focused solely on documents that were cited 110 times or more. The keyword co-occurrence analysis included keywords that appeared in at least 10 publications. Journal impact factor (IF) data were sourced from the 2023 Journal Citation Reports (JCR). The combination of these analytical methods and tools provided a comprehensive and in-depth perspective for understanding the research dynamics and trends related to the MedDiet and DM.

## 3 Results

### 3.1 General landscapes of included documents on MedDiet and DM

**Figure 1:**
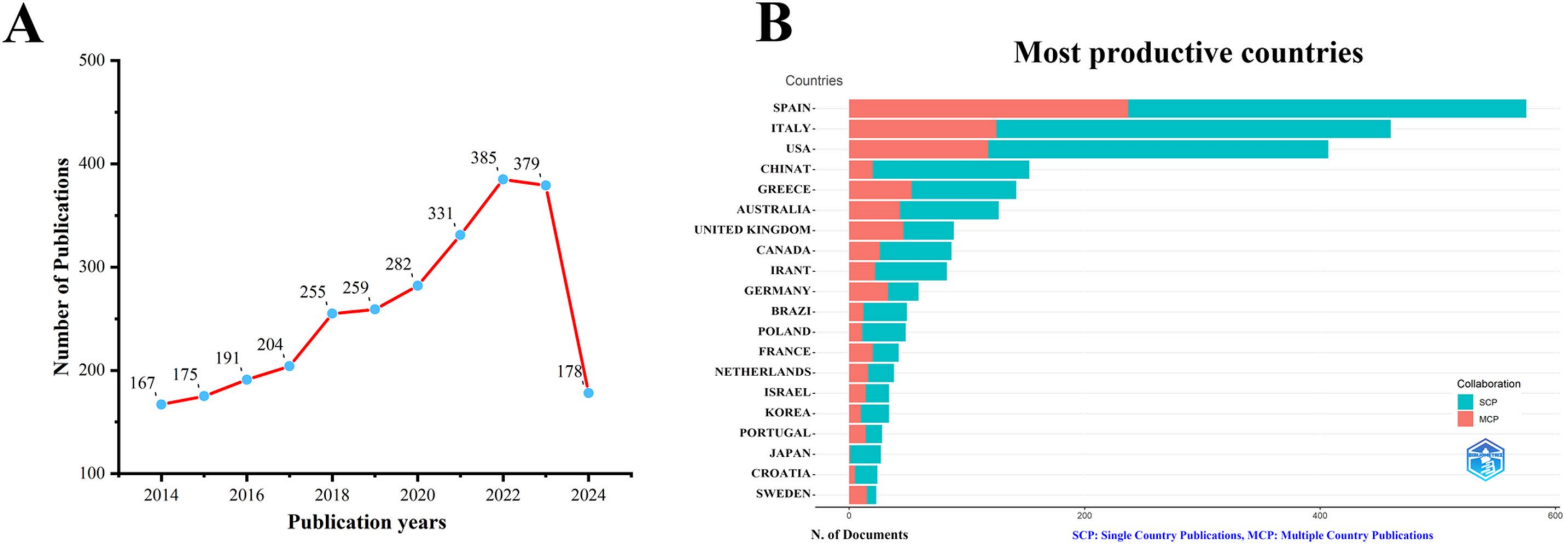
Trends in annual publication outputs the relationship between MedDiet and DM from 2014 to 2024. **(A)** Trends of annual publication outputs. **(B)** Distribution of corresponding authors’ countries and cooperation.

After conducting a search in the WoS database, we identified a total of 2,806 publications related to the MedDiet and DM. As shown in Figure 1A, the number of publications in this field has generally increased over the past decade. From 2014 to 2019, the number of articles published grew slowly, while from 2020 to 2023, there was a rapid rise in publication volume. For 2024, with data available only up to July, the publication count stands at 178. To date, a total of 2,806 articles have been published in this field.

**Figure 2:**
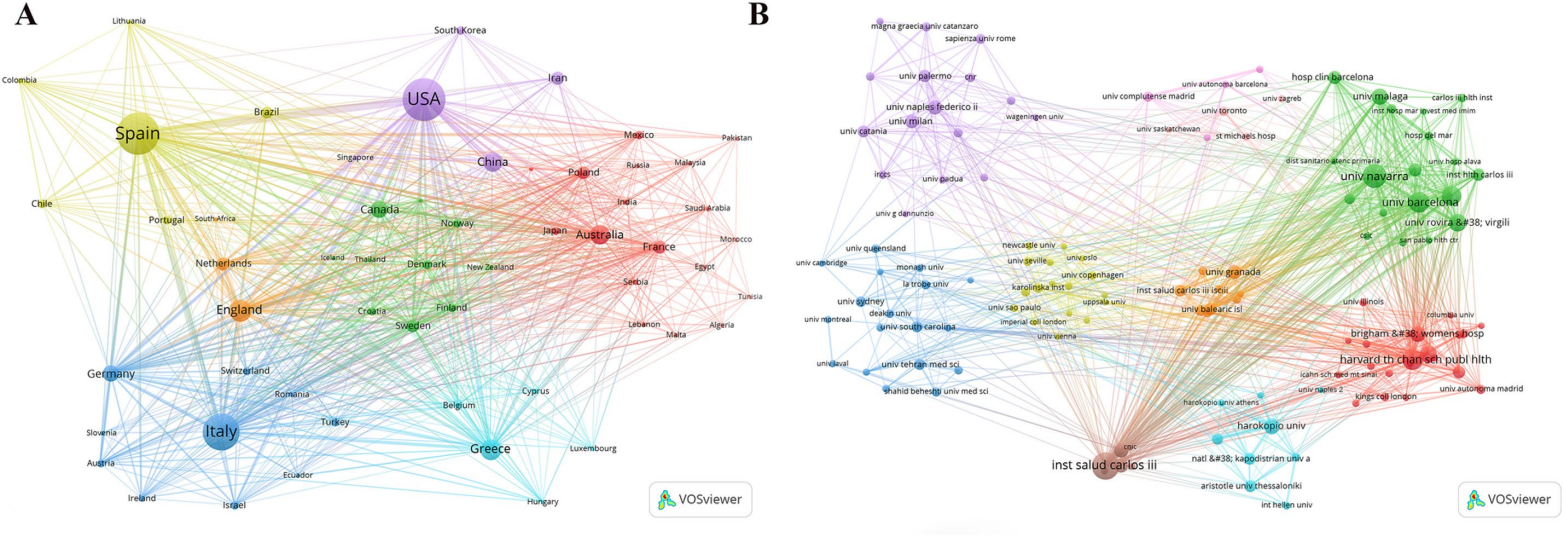
Map of countries/regions and institutions the relationship between MedDiet and DM from 2014 to 2024. **(A)** Map of cooperation between different countries. **(B)** Map of cooperation between different institutions.

Analysis of the corresponding authors’ institutional affiliations by country reveals that Spain (237 articles) leads in research output, followed by Italy (460 articles), the USA (407 articles), China (153 articles), and Greece (142 articles). These results may be related to Spain and Italy, being Mediterranean countries, having a deeper understanding and research focus on the MedDiet. Interestingly, among the top five countries in terms of publication volume, China’s international collaboration rate (13.1%) is significantly lower than that of Spain (41.2%) and Greece (37.3%), indicating less international cooperation in this field from China, as illustrated in Figure 1B and Table 1. Additionally, Figure 2A shows that Spain has the most extensive collaboration with other countries in the field of MedDiet and DM. Furthermore, the collaboration map indicates that Centro de Investigación Biomédica en Red (*n* = 560) and Harvard University (*n* = 557) are prominent centers of collaboration (Figure 2B; Table 2).

**Table 1:** Most relevant countries by corresponding authors of the relationship between MedDiet and DM.

Country	Articles	SCP	MCP	Freq (%)	MCP_Ratio (%)
Spain	575	338	237	20.5	41.2
Italy	460	335	125	16.4	27.2
USA	407	289	118	14.5	29
China	153	133	20	5.5	13.1
Greece	142	89	53	5.1	37.3
Australia	127	84	43	4.5	33.9
United Kingdom	89	43	46	3.2	51.7
Canada	87	61	26	3.1	29.9
Iran	83	61	22	3	26.5
Germany	59	26	33	2.1	55.9
Brazil	49	37	12	1.7	24.5
Poland	48	37	11	1.7	22.9
France	42	22	20	1.5	47.6
Netherlands	38	22	16	1.4	42.1
Israel	34	20	14	1.2	41.2
Korea	34	24	10	1.2	29.4
Portugal	28	14	14	1	50
Japan	27	26	1	1	3.7
Croatia	24	19	5	0.9	20.8
Sweden	23	8	15	0.8	65.2
Mexico	22	13	9	0.8	40.9
Turkey	20	17	3	0.7	15
Chile	18	13	5	0.6	27.8
Spain	575	338	237	20.5	41.2
Italy	460	335	125	16.4	27.2

MCP, multiple-country publication; SCP, single-country publication.

**Table 2:** Most relevant affiliations of the relationship between MedDiet and DM.

Affiliation	Articles (*n*)
Centro de Investigación Biomédica en Red (CIBER)	560
Harvard University	557
Universitat de Barcelona	553
Universidad de Navarra	355
Hospital Clínic de Barcelona	351
CIBER de Fisiopatología de la Obesidad y Nutrición (CIBEROBN)	345
Instituto de Salud Carlos III (ISCIII)	332
Institut d’Investigacions Biomèdiques August Pi i Sunyer (IDIBAPS)	299
Harvard T.H. Chan School of Public Health	290
Universitat Rovira i Virgili	265
Universidad de Málaga	217
Universitat de València	170
Consejo Superior de Investigaciones Científicas (CSIC)	168
Università degli Studi di Napoli Federico II	152
Institut d’Investigació Sanitària Pere Virgili (IISPV)	148
Universidad de Granada	146
Institut Hospital del Mar d’Investigacions Mèdiques (IMIM)	141
Instituto de Investigación Biomédica de Málaga y Plataforma en Nanomedicina (IBIMA)	140
IMDEA Alimentación (IMDEA Food Institute)	138
Universidad de Córdoba	129
University of Toronto	119
Institut d’Investigació Biomèdica de Bellvitge (IDIBELL)	118
National and Kapodistrian University of Athens	118
Hospital del Mar	116
Harvard Medical School	114

### 3.2 Journals and co-cited journals

Using R software (version 3.6.3) with the Bibliometrix and ggplot2 packages, we analyzed the journals with the highest number of publications and the most frequently cited journals in the field of the MedDiet and DM. Additionally, VOSviewer (version 1.6.17) was used for co-citation journal analysis. The analysis revealed that a total of 2,806 articles were published across 668 different academic journals (Annex 1).

Table 3 show that *Nutrients* (*n* = 465, IF = 4.8) is the journal with the highest number of publications, followed by *Nutrition Metabolism And Cardiovascular Diseases* (*n* = 74, IF = 3.3), *Frontiers In Nutrition* (*n* = 69, IF = 4), *British Journal of Nutrition* (*n* = 56, IF = 3), and *American Journal of Clinical Nutrition* (*n* = 54, IF = 6.5).

**Table 3:** Top 10 journals with the most published articles.

Journal	Documents	IF (2023)	Cites
Nutrients	465	4.8	7,413
Nutrition Metabolism and Cardiovascular Diseases	74	3.3	2,176
Frontiers In Nutrition	69	4	487
British Journal of Nutrition	56	3	3,454
American Journal of Clinical Nutrition	54	6.5	7,412
European Journal of Nutrition	51	4.1	1,606
International Journal of Molecular Sciences	45	4.9	1,333
Clinical Nutrition	43	6.6	1,558
Antioxidants	42	6	411
International Journal of Environmental Research And Public Health	37	0	0

Further, Table 4 display the most frequently cited journals, with *Nutrients* (*n* = 7,413, IF = 4.8) leading, followed by *American Journal of Clinical Nutrition* (*n* = 7,412, IF = 6.5), *Diabetes Care* (*n* = 4,403, IF = 6.5), *British Journal of Nutrition* (*n* = 3,454, IF = 3.0), and *New England Journal of Medicine* (*n* = 3,401, IF = 96.2).

**Table 4:** Top 10 journals with the most cited journals.

Journal	Cites	IF (2023)	Document
Nutrients	7,413	4.8	465
American Journal of Clinical Nutrition	7,412	6.5	54
Diabetes Care	4,403	14.8	14
British Journal of Nutrition	3,454	3	56
New England Journal of Medicine	3,401	96.2	1
Journal of Nutrition	3,304	3.7	36
Plos One	3,286	2.9	37
Circulation	3,084	35.5	3
Public Health Nutrition	2,534	3	21
The Lancet	2,447	98.4	0

**Figure 3:**
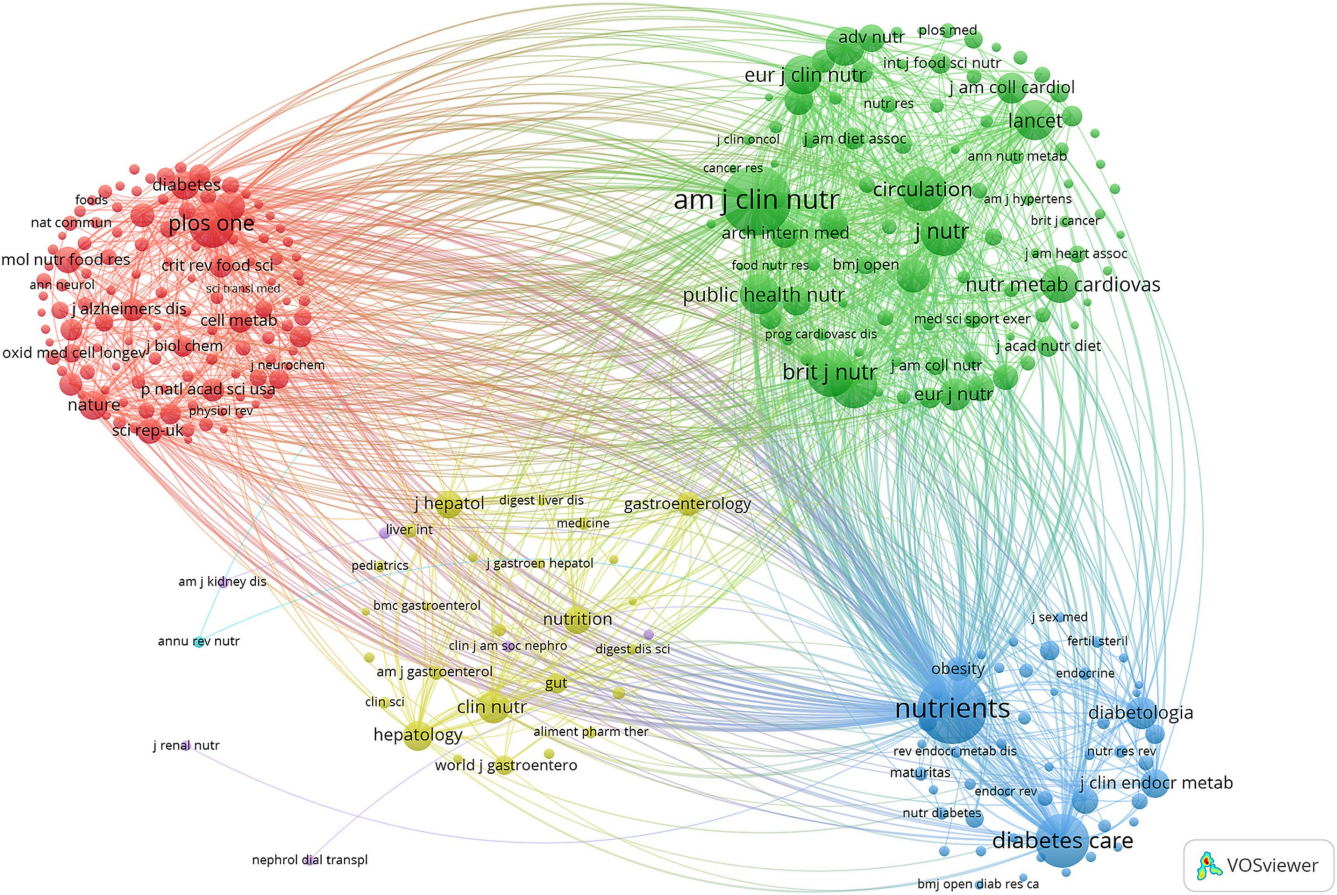
Co-cited journals involved in the relationship between MedDiet and DM.

The co-citation journal visualization analysis (Figure 3) indicates that *Nutrients*, *American Journal of Clinical Nutrition*, and *British Journal of Nutrition* are highly influential journals in this field. These findings highlight the significant roles of *Nutrients* and *American Journal of Clinical Nutrition* in MedDiet and DM research. They also suggest a lack of publications in top-tier journals, which may indicate a need for further improvement in the depth and quality of research.

### 3.3 Most cited references and reference burst

Using the bibliometrix package in R software, we carefully selected the top 20 most-cited articles in the field of MedDiet and DM, all of which have been cited more than 350 times and are scattered across 19 different academic journals (Table 5). This observation suggests that the field has not yet achieved significant theoretical breakthroughs. Notably, none of the journals among these highly cited articles shows a dominant position. The most-cited articles include “Global aetiology and epidemiology of type 2 diabetes mellitus and its complications,” “Primary Prevention of Cardiovascular Disease with a Mediterranean Diet Supplemented with Extra-Virgin Olive Oil or Nuts” and “Global diets link environmental sustainability and human health” which primarily provide comprehensive descriptions of DM and its relationship with dietary habits.

**Table 5:** Top 20 cited references related to the relationship between MedDiet and DM.

Paper	DOI	Total citations	TC per year
ZHENG Y, 2018, NAT REV ENDOCRINOL	10.1038/nrendo.2017.151	3,058	436.86
ESTRUCH R, 2018, NEW ENGL J MED	10.1056/NEJMoa1800389	2,303	329.00
TILMAN D, 2014, NATURE	10.1038/nature13959	1,975	179.55
ROMERO-GÓMEZ M, 2017, J HEPATOL	10.1016/j.jhep.2017.05.016	793	99.13
AUNE D, 2016, BMJ-BRIT MED J	10.1136/bmj.i2716	717	79.67
LEVINE ME, 2014, CELL METAB	10.1016/j.cmet.2014.02.006	607	55.18
DINU M, 2018, EUR J CLIN NUTR	10.1038/ejcn.2017.58	569	81.29
WIDMER RJ, 2015, AM J MED	10.1016/j.amjmed.2014.10.014	511	51.10
SCHWINGSHACKL L, 2017, EUR J EPIDEMIOL	10.1007/s10654-017-0246-y	505	63.13
MARTÍNEZ-GONZÁLEZ MA, 2015, PROG CARDIOVASC DIS	10.1016/j.pcad.2015.04.003	494	49.40
SALAS-SALVADÓ J, 2014, ANN INTERN MED	10.7326/M13-1725	442	40.18
CENA H, 2020, NUTRIENTS	10.3390/nu12020334	416	83.20
SCHWINGSHACKL L, 2017, NUTRIENTS	10.3390/nu9101063	416	52.00
SCHWINGSHACKL L, 2018, J ACAD NUTR DIET	10.1016/j.jand.2017.08.024	411	58.71
TRICHOPOULOU A, 2014, BMC MED	10.1186/1741-7015-12-112	393	35.73
TOSTI V, 2018, J GERONTOL A-BIOL	10.1093/gerona/glx227	380	54.29
KHAZRAI YM, 2014, DIABETES-METAB RES	10.1002/dmrr.2515	365	33.18
GARCIA-MANTRANA I, 2018, FRONT MICROBIOL	10.3389/fmicb.2018.00890	355	50.71
COOPER C, 2015, AM J PSYCHIAT	10.1176/appi.ajp.2014.14070878	354	35.40
ROCK CL, 2020, CA-CANCER J CLIN	10.3322/caac.21591	353	70.60

**Figure 4:**
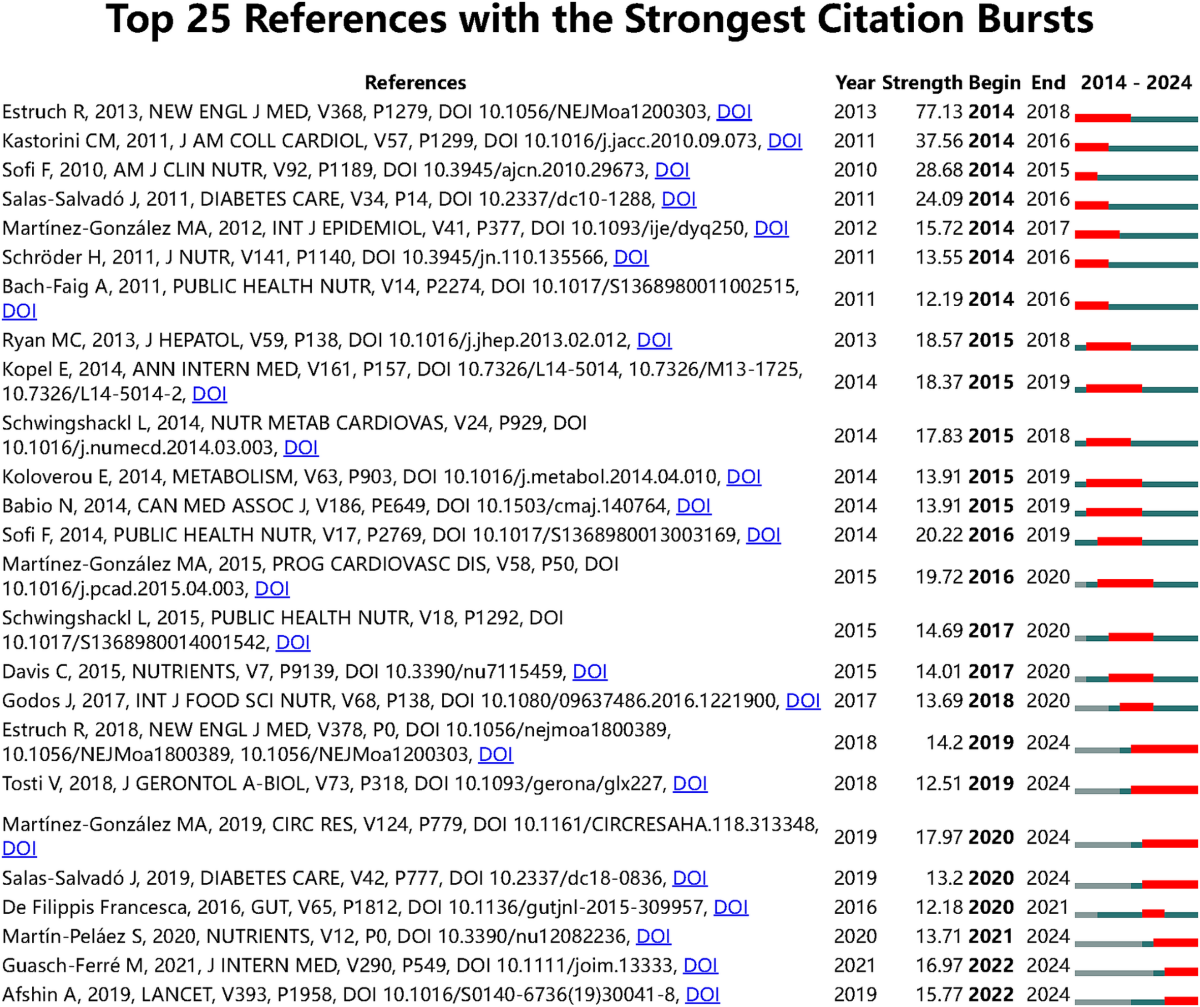
Top 25 references with the strongest citation bursts on the relationship between MedDiet and DM research.

Furthermore, to identify significant citation bursts in the connection between the MedDiet and DM, we used CiteSpace to detect 113 references with notable citation bursts based on specific criteria (top 25; status count: 2; minimum duration: 2), of which 25 are displayed in Figure 4. “Primary Prevention of Cardiovascular Disease with a Mediterranean Diet” leads with an intensity of 77.13, followed by “Meta-Analysis of 50 Studies and 534,906 Individuals” and “Accruing Evidence on Benefits of Adherence to the Mediterranean on Health,” with intensities of 37.56 and 28.68, respectively. It is noteworthy that the three leading citation bursts are “Health Effects of Dietary Risks in 195 Countries, 1990–2017” “Mediterranean Diet Effects on Type 2 DM Prevention” and “Health: A Comprehensive Overview” which provide a broad perspective on the field.

To further understand the research frontiers and hotspots, we matched the DOIs of the 25 citations in Figure 4 with the titles in Annex 2. The results show that 81% of the citations focus on “The Comprehensive Impact of the MedDiet on Chronic Diseases such as Cardiovascular Diseases” 10% are related to the PREDIMED study, and the remaining 10% focus on the measurement and definition of the MedDiet. These data emphasize the importance of the MedDiet in chronic disease prevention, particularly cardiovascular diseases, and highlight the widespread application of the PREDIMED study in this field. However, we also noted that the PREDIMED study may be limited by the dietary habits of volunteers, which could affect their ability to change dietary habits and subsequently impact the accuracy of research results.

### 3.4 Keyword clusters and evolution

Keyword cluster analysis is an effective method for revealing research hotspots and development trends in academic fields, In this study, we used VOSviewer software to extract 4,667 keywords from the literature, According to Table 6, there are 20 keywords that appeared more than 200 times, with “metabolic syndrome” leading with 767 occurrences, followed by “risk” (527), “cardiovascular-disease” (499), “insulin-resistance” (495), “adherence” (329), “association” (291), “meta-analysis” (280), and “obesity” (278).

**Table 6:** Top 20 keywords related to the relationship between MedDiet and DM.

Rank	Words	Occurrences
1	Metabolic syndrome	767
2	Risk	527
3	Cardiovascular-disease	499
4	Insulin-resistance	495
5	Adherence	329
6	Association	291
7	Meta-analysis	280
8	Obesity	278
9	Coronary-heart-disease	273
10	Risk-factors	273
11	Weight-loss	271
12	Physical-activity	249
13	Health	239
14	Blood-pressure	233
15	Prevention	217
16	Oxidative stress	215
17	Disease	192
18	Prevalence	186
19	Patterns	182
20	Mortality	172

**Figure 5:**
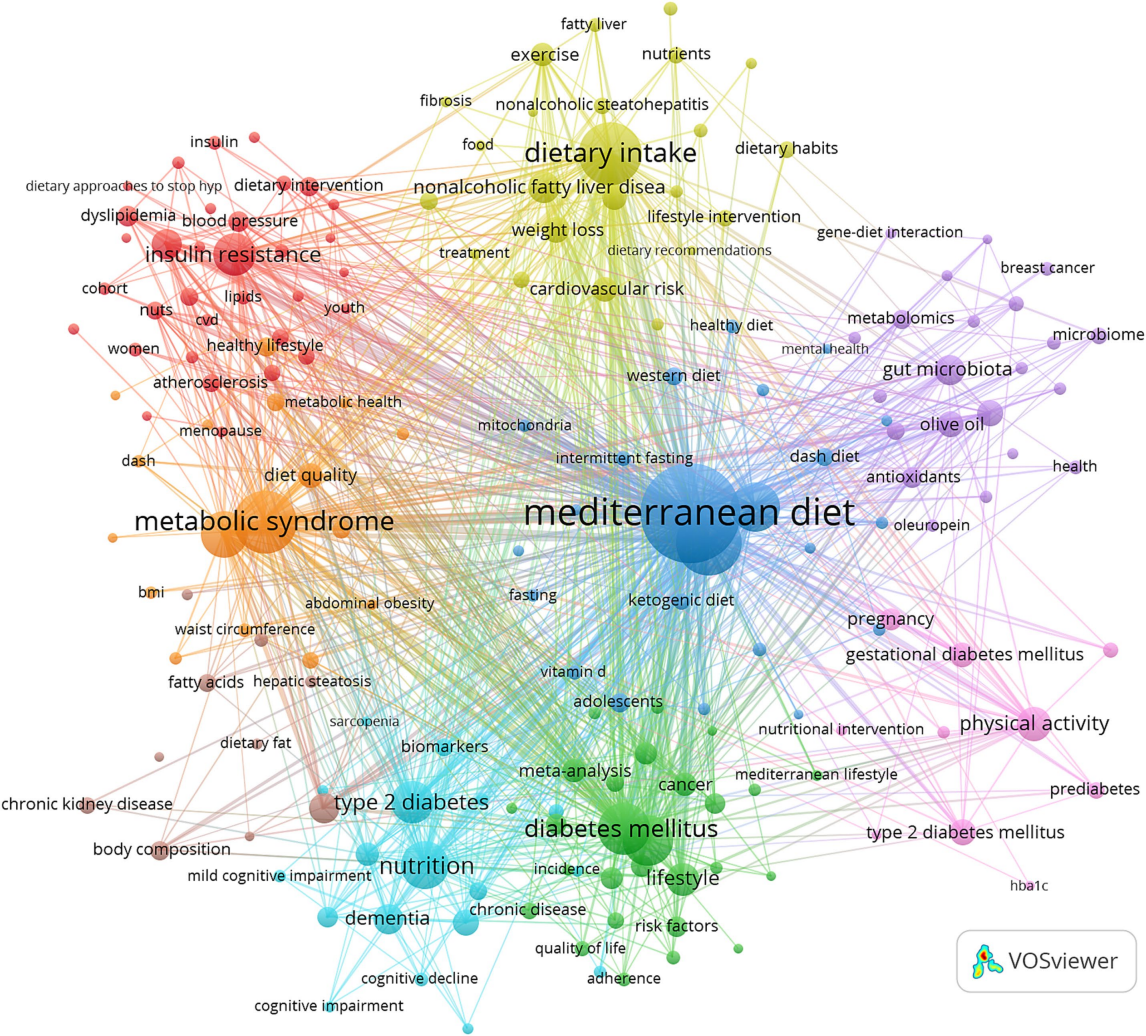
Keyword co-occurrence map of publications on the relationship between MedDiet and DM.

Further, we selected 183 keywords based on the criterion of a minimum occurrence of 10 times and used these to create a keyword cluster map (Figure 5). The map displays nine clusters in different colors as follows.

Cardiometabolic Health Cluster (Red Dot) contains 33 keywords, including adiponectin, adiposity, atherosclerosis, blood pressure and body weight; Chronic Diseases and Prevention Cluster (Green Dot) includes 27 keywords, such as adherence, cancer, cardiovascular diseases, chronic disease and cohort study; Dietary Patterns and Metabolic Health Cluster (Blue Dot) comprises 23 keywords, including adolescents, caloric restriction, cardiovascular risk factors, DASH diet; Nutrition and Lifestyle Interventions Cluster (Yellow Dot) has 23 keywords, such as carbohydrates, cardiovascular risk, dietary habits, exercise and fibrosis; Bioactive Compounds and Personalized Nutrition Cluster (Purple Dot) consists of 22 keywords, including bioactive compounds, epigenetics, lipid metabolism, metabolomics and oleuropein; Aging and Cognitive Health Cluster (Cyan Dot) includes 18 keywords, such as aging, biomarkers, cognition, cognitive function and elderly; Obesity and Metabolic Syndrome Cluster (Orange Dot) contains keywords like abdominal obesity, body mass index, cross-sectional study, MedDiet score, nhanes; Diet and Disease Risk Cluster (Brown Dot) has 10 keywords, including advanced glycation end products, dietary fat, hepatic steatosis, cardiovascular disease risk and predimed; Diabetes and Nutritional Interventions Cluster (Red Dot) includes keywords such as coronavirus disease 2019, SARS-CoV-2 infection, cytokines, gestational diabetes mellitus, nutritional intervention and pregnancy (Annex 3).

**Figure 6:**
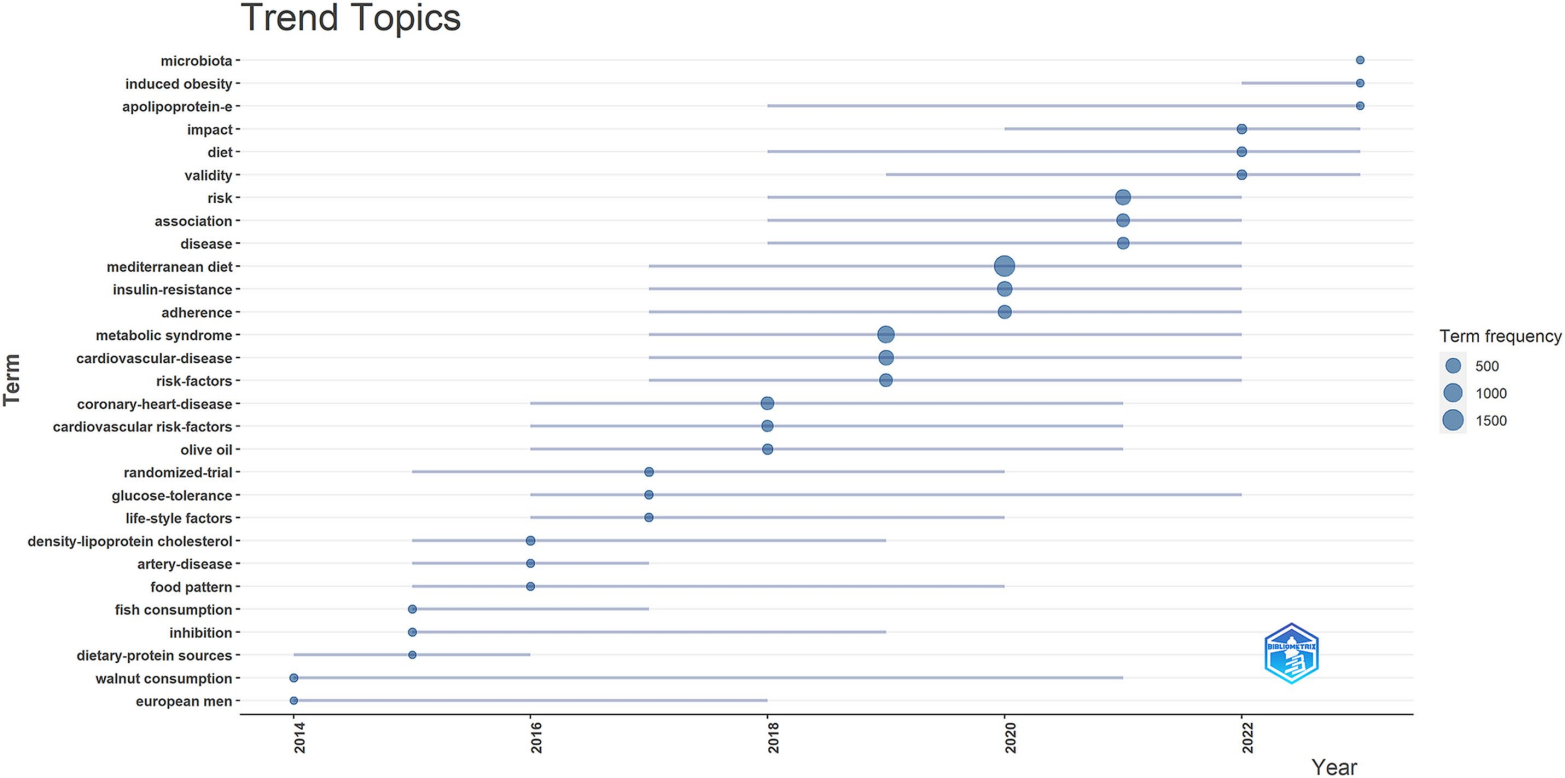
Trend topics on the relationship between MedDiet and DM.

In addition, we utilized the bibliometrix package in R to generate a trend topic map. The trend topic map is a valuable tool for identifying the chronological progression of specific research topics within a given field, allowing us to examine the evolution of research over time. By analyzing the trend topic map shown in Figure 6, we were able to identify the research foci and evolutionary trajectory at different stages within the field. Our findings indicate that the current research in this area is predominantly focused on *microbiota* and *induced obesity*.

## 4 Discussion

### 4.1 General information

In this study, we compiled a comprehensive corpus of 2,806 articles covering the period from 2014 to 2024. By identifying trends and patterns within this corpus, we observed a steady increase in research publications on the interaction between the MedDiet and DM from 2014 to 2023, reflecting a growing interest in this field. In the realm of MedDiet and DM research, Spain has emerged as a leading country, with a significantly higher number of publications. This trend reflects Spain’s historical and cultural connections to the MedDiet, providing a natural advantage and keen interest in related research. Among the 668 journals where 2,806 articles were published, journals such as *Nutrients*, *Nutrition, Metabolism and Cardiovascular Diseases*, and *Frontiers in Nutrition* have played crucial roles. Notably, *Nutrients* has published a substantial number of relevant articles and received extensive citations, establishing itself as a core journal and a key platform for disseminating research findings on the MedDiet and DM.

### 4.2 Hotspots and development trends

Through various analytical methods, including literature clustering, keyword frequency analysis, keyword clustering, and topic evolution, we have identified potential research hotspots related to the use of the MedDiet and DM link. The findings indicate that the forefront and hotspots of research in this field primarily focus on two key aspects. First, the benefits of components in the MedDiet, such as olive oil, legumes, and red wine, for DM. Second, the mechanisms by which the MedDiet prevents DM, including improvements in insulin sensitivity, anti-inflammatory, antioxidant effects, regulation of gut microbiota and fat metabolism.

#### 4.2.1 The benefits of components in the MedDiet, such as olive oil, legumes, and red wine, for DM

Analyzing the articles published in this field, we find that identifying which key components of the MedDiet are beneficial for DM is a current research hotspot and cutting-edge area. The MedDiet is characterized by its high olive oil content, which is the primary source of fat in this diet (32). Moreover, consuming this specific type of olive oil helps reduce the risk of DM (33). Common nuts in the MedDiet, such as almonds, hazelnuts, walnuts, and pistachios, as well as legumes like lentils and chickpeas, are rich in fiber, vitamin B6, magnesium, potassium, and copper (34). These nutrients significantly lower the risk of developing DM (35). The monounsaturated fatty acids (MUFAs) and polyunsaturated fatty acids (PUFAs) found in nuts have been shown to significantly reduce the incidence of DM and its associated cardiovascular diseases (34, 36, 37). Additionally, moderate consumption of red wine, an important component of the MedDiet, has been found to effectively lower the incidence of DM in women (38, 39). Additionally, obesity is a major exacerbating factor for DM, and the MedDiet is superior to low-fat diets in controlling weight and preventing overweight (40–42). These findings provide new perspectives and methods for the prevention and treatment of DM. But it is worth noting that there are still some shortcomings in this kind of research field. (1) Complexity of Components: The analysis in the passage focuses on individual components of the Mediterranean diet, such as olive oil, nuts, and red wine, without considering the potential synergistic or antagonistic effects of these components. The Mediterranean diet is a complex dietary pattern, and interactions between different food components may influence its overall health benefits. Existing research often overlooks these complex interactions, which may lead to an incomplete understanding of the overall benefits of the Mediterranean diet. (2) Generalizability of Findings: The findings mentioned in the passage may be primarily based on specific populations, such as residents of the Mediterranean region or particular gender groups, which could limit their generalizability to different populations globally. For example, the health benefits of the Mediterranean diet may vary among people from different geographical areas, cultural backgrounds, or genetic profiles. Therefore, extrapolating these research findings to all populations may have certain limitations. (3) Consideration of Comorbidities: The research referred to in the passage primarily addresses individual diabetes patients and seldom considers populations with diabetes combined with other conditions. For instance, while the Mediterranean diet recommends moderate red wine consumption, for some patients, consuming red wine may lead to other health issues.

#### 4.2.2 Research on the mechanisms by which the MedDiet prevents DM

In research on the mechanisms by which the MedDiet influences DM, analysis of keywords and citation literature reveals that common research mechanisms in this field include anti-inflammatory and antioxidant effects, regulation of gut microbiota, improvement of insulin sensitivity and secretion, and enhancement of fat metabolism. Oxidative stress is a pathological state caused by an imbalance between the excessive generation of reactive oxygen species (ROS) and the body’s antioxidant defenses (43). ROS, including free radicals and other highly reactive molecules, are byproducts of normal cellular metabolism; however, excessive levels can impair β-cell function, lead to insulin resistance, and disrupt glucose regulation (44). Moreover, oxidative stress is closely related to DM-associated retinopathy and cardiovascular diseases (45, 46). Research indicates that inflammation is also a significant factor in inducing insulin resistance, which contributes to the development of DM (47).

Analysis of published literature in this field shows that the Mediterranean diet is rich in antioxidants, such as polyphenols, plant sterols, and carotenoids, which inhibit oxidative stress and inflammatory responses through various molecular mechanisms. For example, epigallocatechin-3-gallate (EGCG) in green tea reduces inflammation and oxidative stress by inhibiting the NF-kB pathway (48). Lycopene improves type 2 diabetes by activating antioxidant systems like superoxide dismutase (SOD) and glutathione peroxidase (49). In the same vein, leafy greens and nuts rich in vitamin E protect cell membranes by activating NRF2 and heat shock proteins, reducing ROS damage to cells, and inhibiting NF-kB activity, thereby alleviating oxidative stress in DM patients (50).

The gut microbiota plays a crucial role in human health, with its dysregulation closely linked to various diseases, including DM (51, 52). Recent studies suggest that the MedDiet increases the abundance of Roseburia in the gut, a microbiota negatively associated with DM (53, 54). The MedDiet provides abundant fermentable substrates, promoting the production of short-chain fatty acids (SCFAs) (55). SCFAs help improve insulin secretion, reduce insulin resistance, suppress glucose production, and decrease food intake by regulating the secretion of hormones like GLP-1, leptin, and ghrelin, thereby playing a positive role in DM prevention and management (56).

The MedDiet emphasizes extra-virgin olive oil as its primary fat source and promotes moderate consumption of animal proteins and fats, favoring fish, eggs, and low-fat dairy products. Recent studies suggest that strong adherence to the MedDiet is significantly linked to reductions in both fasting and postprandial insulin resistance in individuals with overweight or obesity, with fish consumption being a key contributor to this effect (57). Fish is rich in omega-3 fatty acids, which offer various metabolic benefits that enhance insulin sensitivity and reduce the risk of type 2 diabetes mellitus. Moreover, a recent meta-analysis of 46 randomized clinical trials involving omega-3 fatty acids (*n* = 4,991 patients with type 2 diabetes) demonstrated improvements in HbA1c levels (58). The beneficial effects of omega-3 fatty acids can be attributed to multiple mechanisms. Notably, these fatty acids inhibit lipolysis, thereby limiting the release of free fatty acids into the bloodstream and alleviating their interference with insulin signaling pathways (59). Furthermore, omega-3 fatty acids stimulate mitochondrial biogenesis, enhance the expression of genes involved in fatty acid oxidation, and suppress lipid synthesis, which collectively decrease fat accumulation in adipose tissue (60). In addition, their anti-inflammatory properties boost the production of adiponectin, an adipokine that plays a crucial role in improving insulin sensitivity (61). In summary, the diverse actions of omega-3 fatty acids significantly contribute to metabolic health and help lower the risk of type 2 diabetes mellitus.

In fact, our analysis of the literature reveals that many mechanisms underlying the MedDiet benefits for DM patients remain inadequately understood. While there are indications of beneficial effects, the specific molecular mechanisms are not fully clear. The interplay between oxidative stress and inflammatory responses, as well as the mechanisms by which the MedDiet influences DM through gut microbiota and regulation of fat metabolism, have only been superficially explored. Therefore, future research should focus on analyzing the interactions between these mechanisms, with particular emphasis on gut microbiota, to enhance our understanding of the molecular pathways involved.

### 4.3 Limitations

This study utilized the WoSCC database as a data source, providing a comprehensive overview of the field, including its overall scope, hotspots, and research trends. This approach aids in a deeper understanding of the field and helps explore future research direction. However, there are several notable limitations to this study.

First, reliance solely on the WoSCC database may result in the omission of some relevant publications, despite the database’s high quality and widespread recognition as an ideal tool for bibliometric analysis (62–64). Second, the study only includes English-language publications, which may introduce language bias and limit the generalizability of the findings. Third, due to the presence of many authors with the same name in China, the study did not conduct an in-depth analysis of authors to avoid potential misleading results. Despite these limitations, the conclusions of this study remain highly reliable and provide valuable insights and references for academic research in the field.

## 5 Conclusion

This study systematically reveals the major research hotspots and frontiers in the relationship between the MedDiet and DM, summarized as follows:

- Research on the association between the MedDiet and DM has garnered widespread attention from scholars globally. Spain, Italy, the United States, China, and Greece are the most active countries in this field, with close and extensive international research collaborations.
- *Nutrients* and *Nutrition, Metabolism & Cardiovascular Diseases* are the most prolific journals in this area. Notably, *Nutrients* stands out as a representative journal due to its high citation rate.
- Components such as olive oil, legumes, and red wine are key research hotspots and trends in the prevention and management of DM within the MedDiet.
- Core mechanisms of interest in the relationship between the MedDiet and DM include anti-inflammatory and antioxidant effects, improvement of insulin sensitivity and secretion, and regulation of fat metabolism. Additionally, the “gut microbiota” is an emerging area of research that is receiving increasing attention.

In summary, this study provides in-depth and valuable insights into the research trends and hotspots concerning the MedDiet and DM. These findings not only offer researchers essential background knowledge for a comprehensive understanding of the field but also lay the foundation for exploring new research directions. By identifying current research frontiers and potential key areas, our study provides strong support and guidance for future innovative research.

## Data Availability

Publicly available datasets were analyzed in this study. This data can be found here: Web of Science database (https://www.webofscience.com/).
