# Supplementary material for: Global research dynamics in the Mediterranean diet and diabetes mellitus: a bibliometric study from 2014 to 2024
Source: Front Nutr. 2024 Nov 14;11:1480856. doi: 10.3389/fnut.2024.1480856 (PMC11603804; doi:10.3389/fnut.2024.1480856)
Supplement: Supplementary file 3 [file Table_3.DOCX]

Cluster1 (33)

adiponectin

adiposity

atherosclerosis

blood pressure

body weight

cardiometabolic risk

children

cholesterol

cohort

cvd

dietary approaches to stop hypertension

dietary intervention

dyslipidemia

endothelial function

glucose

glycemic control

hyperglycemia

hypertension

inflammatory markers

insulin

insulin resistance

insulin sensitivity

lipid profile

lipids

mediterranean diet adherence

mediterranean dietary pattern

menopause

nuts

pediatric obesity, juvenile obesity

type 1 diabetes

weight

women

youth

cluster2 (27)

adherence

cancer

cardiovascular diseases

chronic disease

chronic diseases

cohort study

coronary heart disease

depression

diabetes mellitus

epidemiology

gender

heart failure

incidence

lifestyle

mediterranean

mediterranean lifestyle

meta-analysis

myocardial infarction

nordic diet

primary prevention

public health

quality of life

review

risk factors

secondary prevention

stroke

systematic review

cluster3 (23)

adherence

cancer

cardiovascular diseases

chronic disease

chronic diseases

cohort study

coronary heart disease

depression

diabetes mellitus

epidemiology

gender

heart failure

incidence

lifestyle

mediterranean

mediterranean lifestyle

meta-analysis

myocardial infarction

nordic diet

primary prevention

public health

quality of life

review

risk factors

secondary prevention

stroke

systematic review

cluster3 (23)

| adolescents |
| --- |
| caloric restriction |
| cardiovascular risk factors |
| dash diet |
| dietary patterns |
| fasting |
| healthy diet |
| high-fat diet |
| intermittent fasting |
| ketogenic diet |
| low-carbohydrate diet |
| low-fat diet |
| mediterranean diet |
| mental health |
| metabolic disorders |
| mitochondria |
| overweight |
| plant-based diet |
| polycystic ovary syndrome |
| sleep |
| vegetarian diet |
| vitamin d |
| western diet |

Cluster4 (23)

| carbohydrates |
| --- |
| cardiovascular risk |
| dietary habits |
| dietary intake |
| dietary recommendations |
| exercise |
| fatty liver |
| fibrosis |
| food |
| lifestyle intervention |
| macronutrients |
| metabolism |
| microbiota |
| micronutrients |
| nafld |
| nash |
| non-communicable diseases |
| nonalcoholic fatty liver disease |
| nonalcoholic steatohepatitis |
| nutrients |
| steatosis |
| treatment |
| weight loss |

Cluster5 (22)

| antioxidants |
| --- |
| bioactive compounds |
| breast cancer |
| cardiovascular |
| epigenetics |
| extra virgin olive oil |
| gene-diet interaction |
| gut microbiota |
| health |
| hydroxytyrosol |
| lipid metabolism |
| metabolomics |
| microbiome |
| nutrigenetics |
| nutrigenomics |
| oleuropein |
| olive oil |
| personalized nutrition |
| phenolic compounds |
| polyphenols |
| precision nutrition |
| resveratrol |

Cluster6 (18)

| aging |
| --- |
| biomarkers |
| cognition |
| cognitive decline |
| cognitive function |
| cognitive impairment |
| dementia |
| elderly |
| frailty |
| legumes |
| mild cognitive impairment |
| nutrition |
| older adults |
| prevention |
| probiotics |
| sarcopenia |
| type 2 diabetes |
| vegetables |

Cluster7 (17)

| abdominal obesity |
| --- |
| bmi |
| body mass index |
| c-reactive protein |
| cross-sectional study |
| dash |
| diet quality |
| dietary inflammatory index |
| erectile dysfunction |
| healthy lifestyle |
| inflammation |
| mediterranean diet score |
| metabolic health |
| metabolic syndrome |
| mortality |
| nhanes |
| waist circumference |

Cluster8 (10)

| advanced glycation end products |
| --- |
| body composition |
| cardiovascular disease risk |
| chronic kidney disease |
| dietary fat |
| fatty acids |
| glycemic index |
| hepatic steatosis |
| oxidative stress |
| predimed |

Cluster9 (10)

| coronavirus disease 2019, sars-cov-2 infection |
| --- |
| cytokines |
| gestational diabetes mellitus |
| hba1c |
| nutritional intervention |
| physical activity |
| prediabetes |
| pregnancy |
| randomized controlled trial |
| type 2 diabetes mellitus |
